# A classical test theory evaluation of the Sleep Condition Indicator accounting for the ordinal nature of item response data

**DOI:** 10.1371/journal.pone.0213533

**Published:** 2019-03-14

**Authors:** Amanda Hellström, Peter Hagell, Anders Broström, Martin Ulander, Annemarie I. Luik, Colin A. Espie, Kristofer Årestedt

**Affiliations:** 1 Faculty of Health and Life Sciences, Linnaeus University, Kalmar, Sweden; 2 The PRO-CARE Group, Faculty of Health Sciences, Kristianstad University, Kristianstad, Sweden; 3 School of Health and Welfare, Jönköping University, Jönköping, Sweden; 4 Department of clinical neurophysiology, Linköping University hospital, Linköping, Sweden; 5 Division of Neurosciences and Inflammation Research, Department of Clinical and Experimental Medicine, Linköping University, Linköping, Sweden; 6 Sleep and Circadian Neuroscience Institute, Nuffield Department of Clinical Neurosciences, University of Oxford, Oxford, United Kingdom; 7 Department of Epidemiology, Erasmus MC University Medical Center, Rotterdam, Netherlands; 8 The Research Section, Region Kalmar County, Kalmar, Sweden; University of Zaragoza, SPAIN

## Abstract

**Background:**

Insomnia symptoms are common among young adults and affect about 5% to 26% of 19 to 34-year-olds. In addition, insomnia is associated with poor mental health and may affect daily performance. In research, as well as in clinical practice, sleep questionnaires are used to screen for and diagnose insomnia. However, most questionnaires are not developed according to current DSM-5 diagnostic criteria. An exception is the recently developed Sleep Condition Indicator (SCI), an eight-item scale screening for insomnia.

**Aim:**

The aim of this study was to perform a Classical Test Theory (CTT) based psychometric evaluation of the SCI in a sample of Swedish university students, by taking the ordinal nature of item level data into account.

**Methods:**

The SCI was translated into Swedish and distributed online to undergraduate students at three Swedish universities, within programs of health, psychology, science or economy. Of 3673 invited students, 634 (mean age 26.9 years; SD = 7.4) completed the questionnaire that, in addition to the SCI, comprised other scales on sleep, stress, lifestyle and students’ study environment. Data were analyzed according to CTT investigating data completeness, item homogeneity and unidimensionality.

**Results:**

Polychoric based explorative factor analysis suggested unidimensionality of the SCI, and internal consistency was good (Cronbach’s alpha, 0.91; ordinal alpha, 0.94). SCI scores correlated with the Insomnia Severity Index (-0.88) as well as with sleep quality (-0.85) and perceived stress (-0.50), supporting external construct validity.

**Conclusions:**

These observations support the integrity of the of the SCI. The SCI demonstrates sound CTT-based psychometric properties, supporting its use as an insomnia screening tool.

## Introduction

Insomnia disorder is defined as difficulty initiating or maintaining sleep and/or waking up too early, accompanied by daytime complaints for at least 3 days a week for 3 or more months [1]. Insomnia is associated with a range of health problems, such as cardiovascular disorders and type 2 diabetes, and mental disorders such as depression, anxiety, bipolar disorder and suicidal ideation [2, 3]. There is a preponderance of insomnia in women compared to men [4, 5] and there is an age-related increase in prevalence [6]. Insomnia symptoms have been reported in 5.3% - 26.3% of young adults (19–34 years) in the general population [4], with a somewhat higher rate (9.5% - 39.4%) among university students [7, 8].

There is a need for valid insomnia screening tools to identify people with sleep problems. Several screening tools have been proposed, e.g. the Athens Insomnia Scale [9], the Minimal Insomnia Symptom Scale [10] and the Insomnia Severity Index [11]. However, these were developed prior to publication in 2013 of the updated, 5^th^ version of the Diagnostic and Statistical Manual of Mental Disorders (DSM-5) [1]. This revision incorporates updated frequency criteria, an extended duration of symptoms, and a departure from the previous distinction between primary and secondary insomnia. Criteria for chronic insomnia within the International Classification of Sleep Disorders (ICSD-3) [12] are largely in line with the DSM-5.

The recently developed Sleep Condition Indicator (SCI) is a clinical screening instrument based on the DSM-5 criteria for insomnia [13]. The SCI is now widely used in clinical practice, and studies have been published providing referent sex and age population values [6, 14]. The SCI was developed and evaluated using classical test theory (CTT) in the United Kingdom. The original version showed high internal consistency (Cronbach’s alpha 0.86) and a two-factor structure has been suggested based on a principal component analysis, were items 1, (getting to sleep); 2, (remaining asleep); 3, (nights per week); 4, (sleep quality) and 8, (duration of problem) loaded strongest on the first factor and items 5, (personal functioning); 6, (daytime performance) and 7, (troubled or not) on the second factor [6, 13]. Subsequently, the SCI has been translated and tested in Italy [15], France [16] and Hong Kong [17] again using traditional classical test theory (CTT) methodology.

However, traditional correlational-based CTT (e.g., Cronbach’s coefficient alpha, corrected item-total correlations and exploratory factor analysis) is commonly based on parametric statistics that do not take the ordinal nature of item level data into account. In addition, item level data are often skewed. Taken together, these features can result in biased estimates. For example, the underlying assumptions of traditional correlations generally tend to deflate reliability estimates such as coefficient alpha. Similarly, factor analyses tend to suggest factors that are artifacts of item difficulty rather than reflecting underlying constructs [18–20]. Such biases are of relevance for researchers as well as for sleep clinicians, because of the implications they have for the use of the SCI and the interpretation of collected data.

The aim of this study was therefore to perform a CTT based psychometric evaluation of the SCI in a sample of Swedish university students, by taking the ordinal nature of item level data into account.

## Method

### Design

This psychometric evaluation was embedded in a study investigating sleep and its associations with the lifestyle and study situation of university students. The study was approved by the Regional Ethical Review Board in Linköping (No. 2016/146-31).

### Participants

Participants were recruited from three middle-sized Swedish universities with 11,000 to 34,000 full-time students. Selection criteria were full-time Swedish speaking undergraduate students admitted to programs within health, psychology, science or economics, with a valid email address. In total, 3,673 students were invited to participate in this online survey. Approval and access to valid email addresses were gained through the responsible deans at each university. After the initial invitation, three e-mail reminders were sent to non-responders over a period of six weeks. The web questionnaire was open for 66 days. Consent to participation was confirmed by a completed questionnaire, which was clearly stated in the information the students received before starting the web questionnaire.

### The survey

The online web survey contained questions that yielded demographic data such as sex, age, self-rated health, and questionnaires regarding sleep, perceptions about their studies, perceived stress, self-efficacy and lifestyle factors. For this project, data was obtained by the questionnaires as described below.

#### Sleep Condition Indicator (SCI)

The SCI comprises eight items relevant to the evaluation and screening of insomnia in clinical practice. The items are responded to according to five ordered response categories scored 0–4, with higher scores indicating better sleep. A summed total score (possible range, 0–32) is then calculated and a score of ≤16 has been suggested to identify people who likely suffer from insomnia [13]. The SCI is informed by the DSM-5 criteria for insomnia disorder, including daytime factors, which are important drivers of clinical symptoms needed for an insomnia diagnosis. Finally, the SCI provides a dimensional perspective on sleep, on a global scale where higher scores represent better sleep [13]. There are similarities to the more established Insomnia Severity Index (ISI) [11] although the SCI could be seen as more contemporary in relation to insomnia diagnostic criteria.

As no Swedish version of SCI was available, it was translated according to a forward-backward procedure [21]. First, two independent native Swedish bilingual translators translated the original UK English version into Swedish. Both translated versions were then compared by the researchers and merged into one version. In a third step, two other independent bilingual native UK English speakers, back-translated the Swedish SCI into UK English. Finally, the two back-translations were compared to the original UK English SCI. Before the final Swedish version was determined, the original constructor was contacted to discuss minor deviations between the original and back translated version. This did not result in any changes.

The Swedish SCI was then assessed using cognitive interviews [22] in a sample of ten undergraduate and two doctoral students. All participants were interviewed by the first author (AH) and gave permission to be audio recorded during the interview. Respondents were requested to think aloud while going through the questionnaire [22]. The interviews aimed to evaluate clarity and relevance of the translated questionnaire. All interviewees were women (median age = 23.5 years, range = 19–64). The mean time needed to complete the SCI was 2 (range = 1–3) minutes. Overall, participants perceived the SCI as easy to understand and the instructions as straightforward and comprehensive. Items 5 (personal functioning), 6 (daytime performance) and 7 (troubled or not) were perceived as closely related but nonetheless reflecting different aspects of daytime dysfunction. Some respondents found the response categories ‘a little’ and ‘somewhat’ as difficult to distinguish between. Item 8 (duration of problem) was perceived as focusing on those having difficulties with sleep and could be considered less relevant to people without sleep difficulties. Some respondents reported on recurring periods of poor sleep that could not be detected by item 8, since they currently slept well. Respondents also asked for an item on sleep duration. However, since none of the comments could be related to issues in the translation, no changes were made based on the cognitive interviews.

#### Insomnia Severity Index (ISI)

The ISI contains seven items about how a person is affected by sleep difficulties and what kind of difficulties he/she has experienced during the last two weeks [11]. A total score (0–28) is calculated and a higher score indicates more insomnia symptoms. Ordinal alpha of the ISI in the present study was 0.93.

#### Pittsburgh Sleep Quality Index (PSQI)

The PSQI is an 18-item questionnaire regarding sleep quality in the last month, covering aspects such as sleep onset latency, sleep efficiency, usage of hypnotics and effects on daytime performance [23]. Although not specifically designed as an insomnia questionnaire, sleep quality is highly related to insomnia [23]. The PSQI yields a total score in the range 0–21 where higher scores indicate poorer sleep quality. A score of >5 has been suggested to represent poor sleep quality. Ordinal alpha of the PSQI in the present study was 0.83.

#### Perceived Stress Scale (PSS)

The 14-item PSS assesses the degree to which people have perceived their lives as stressful in the last month. Each item is scored from 0–4. Seven items are positively worded and seven are negatively worded. Scoring of the negative items is reversed before calculation of a total score (possible range, 0–56), so that a higher score suggest more stress [24]. Ordinal alpha of the PSS in the present study was 0.89.

### Analyses

Data were analyzed according to CTT [25, 26] using Stata 15.1 (Stata Corp., College Station, TX, USA), Factor 10.4, (Rovira i Virgili University, Tarragona, Spain), R 3.4.2 (The R Foundation for Statistical Computing, Vienna, Austria) using the psych package (version 1.7.8) and SPSS 24.0 (IBM Corp., Armonk, NY, USA).

Data completeness was evaluated by the percentage of missing item responses, which should be <10% [27]. Targeting, i.e. how well scale scores are in agreement with levels of insomnia, was assessed through score distributions, skewness and floor-/ceiling effects. A well-targeted scale should, as a rule of thumb, have an average score close to the scale midpoint and span most of its potential range, without excess skewness (preferable between -1 and +1), and with floor/ceiling effects not exceeding 20% [25]. In addition, the patterns of item endorsement frequencies were examined as well as item level endorsement distributions across response categories.

Homogeneity across items was evaluated using polyserial correlations. Inter-item correlations should be between 0.3 to 0.7. Values below 0.3 suggest low congruence with the underlying construct, while values above 0.7 suggest redundancy [28].

Exploratory factor analysis (EFA) was used to evaluate the dimensionality of the SCI. To take the ordinal nature of item level data into account, the EFA was based on a polychoric correlation matrix, using unweighted least square (ULS) extraction (Kaiser-Meyer-Olkin test = 0.91; Bartlett's test of sphericity χ^2^ (28)). A parallel analysis (based on 500 random permutations) was used to decide number of factors instead of the Kaiser criterion, which is known to yield biased results [29]. Finally, two different goodness of fit statistics were used to examine the final model; root mean square error of approximation (RMSEA; should be below 0.05) and comparative fit index (CFI; should be above 0.95) [30].

Considering the ordinal data, internal consistency was estimated using ordinal alpha, based on polychoric correlations. Ordinal alpha values can be interpreted in the same way as traditional Cronbach’s alpha, and should be at least >0.7 and preferably >0.8 [18]. Cronbach’s alpha was computed as well.

To evaluate external construct validity, SCI scores were correlated with ISI, PSQI and PSS scores using the Spearman’s rho correlation coefficient (r_s_). It was hypothesized that SCI scores should correlate strongly (r_s_ >0.8) with ISI and PSQI scores as both are sleep-related constructs. Perceived stress has previously been associated with sleep quality in university students [31]. The PSS was therefore included in the survey as a related construct, associated with quality of sleep rather than aspects of quantity [32], and it was hypothesized that PSS scores would show a weaker correlation with SCI scores than with ISI and PSQI scores.

A receiver operating characteristic (ROC) curve was used to evaluate the discriminating abilities of the SCI. The area under curve (AUROC) provides a value of the ability to discriminate good sleepers from poor sleepers. If the curve covers the whole diagram, the area is 1, which represents a perfect test. The area should be >0.50 in order for a test to be useful. Furthermore, Youden's index (J = sensitivity + specificity—1) was used to evaluate the cut-off score, since both specificity and sensitivity were considered as equally important. A perfect test yields a value of 1 on the Youden index, therefore the cut-off associated with the highest Youden index is considered the optimal [33]. Since we lacked an clinical interview to diagnose insomnia, we used the ISI as a proxy. A score of 15 or higher on ISI was considered insomnia disorder [11]. Before making the calculation, the scores on ISI was reversed, in order to match the SCI (where lower score means poorer sleep).

## Results

### Characteristics of survey responders

The final sample consisted of 634 students with a mean age of 26.9 (SD = 7.4) years, representing a response rate of 17.3%. The majority of participants was female (83.1%) and more than half of the sample were nursing students (Table 1). We found no statistically significant differences in sleep problems between students in the different educational programs in the study.

**Table 1:** Description of the study sample (n = 634).

Age, years (n = 629), mean (SD)	26.9 (7.4)
**Gender, n (%)**	
Female	527 (83.1)
Male	106 (16.7)
Other gender identity	1 (0.2)
**Living situation, n (%)**	
Living alone	237 (37.4)
Co-habiting	393 (62.0)
Missing data	4 (0.6)
**Programs, n (%)**	
Biology	9 (1.4)
Dental hygiene	29 (4.6)
Economy	44 (6.9)
Environmental analytics	22 (3.5)
Medical Laboratory Science	36 (5.7)
Nursing	388 (61.2)
Optics	6 (0.9)
Occupational therapy	14 (2.2)
Pharmacology	23 (3.6)
Psychology	28 (4.4)
Health Science	27 (4.3)
Nutrition and Food science	5 (0.8)
Missing data	3 (0.5)
**General health, n (%)**	
Poor	18 (2.8)
Fair	139 (21.9)
Good	262 (41.3)
Very good	176 (27.8)
Excellent	38 (6.0)
Missing data	1 (0.2)
**Perceived Stress Scale, PSS,** (n = 593), median (q1-q3)	27 (21–33)
**Sleep Condition Indicator, SCI,** (n = 614), median (q1-q3)	23 (15–28)
**Insomnia Severity Index, ISI,** (n = 590), median (q1-q3)	7 (3–13)
**Pittsburgh Sleep Quality Index, PSQ,** (n = 548), median (q1-q3)	6 (4–9)

PSS (score range, 0–56; higher scores indicate higher level of stress); SCI (score range, 0–32; score ≤16 indicates sleep difficulties); ISI (score range, 0–28, scores >14 indicates sleep difficulties); PSQI (score range, 0–21, scores >5 indicates poor sleep quality)

### Psychometric testing of the Sleep Condition Indicator

Data completeness was excellent. Missing data varied between 0.2% and 1.3% across items and 96.9% of all respondents had no missing data (Table 2).

**Table 2:** Item endorsement frequencies of the Sleep Condition Indicator (n = 634).

	Item score distribution, n (%)					
Items^1^	4	3	2	1	0	Missing
Thinking about a typical night in the last month… [Tänk på en typisk natt den senaste månaden…]^2^						
	**0–15 min**	**16–30 min**	**31–45 min**	**46–60 min**	**>60 min**	
1 …how long does it take you to fall asleep? Swe:…hur lång tid tar det för dig att somna på kvällen?	227 (35.8)	212 (33.4)	93 (14.7)	49 (7.7)	51 (8)	2 (0.3)
2 …if you then wake up during the night… how long are you awake for in total? (add all wakenings up) […om du vaknar på natten, hur länge är du då vaken totalt? (Lägg ihop samtliga vakna stunder)]	370 (58.4)	128 (20.2)	48 (7.6)	47(7.4)	40 (6.3)	1 (0.2)
	**0–1 nights**	**2 nights**	**3 nights**	**4 nights**	**5–7 nights**	
3 …how many nights a week do you have a problem with your sleep? […hur många nätter i veckan har du problem med din sömn?]	330 (52.1)	92 (14.5)	73 (11.5)	55(8.7)	81 (12.8)	3 (0.5)
	**Very good**	**Good**	**Average**	**Poor**	**Very poor**	
4 …how would you rate your sleep quality? […hur skulle du bedöma din sömnkvalitet?]	119 (18.8)	248 (39.1))	149 (23.5)	94 (14.8)	22 (3.5)	2 (0.3)
Thinking about the past month, to what extent has poor sleep…						
[Med den senaste månaden i åtanke, hur mycket har dålig sömn…]	**Not at all**	**A little**	**Somewhat**	**Much**	**Very much**	
5 …affected your mood, energy or relationships? […din sinnesstämning, ork eller relationer till andra?]	103 (16.2)	241 (38.0)	158 (24.9)	87 (13.7)	42 (6.6)	3 (0.3)
6 …affected your concentration, productivity, or ability to stay awake? […påverkat din koncentrationsförmåga, produktivitet eller förmåga att hålla dig vaken?]	101 (15.9)	230 (36.3)	162 (25.6)	98 (15.5)	38 (6.0)	5 (0.8)
7 …troubled you in general? […besvärat dig i allmänhet?]	134 (21.1)	253 (39.9)	139 (21.9)	68 (10.7)	34 (5.4)	6 (0.9)
	**I don’t have a problem /<1 mo**	**1–2 mo**	**3–6 mo**	**7–12 mo**	**>1 yr**	
8 Finally… how long have you had a problem with your sleep? [Slutligen…hur länge har du haft problem med din sömn?]	313 (49.4)	61 (9.6)	48 (7.6)	28 (4.4)	176 (27.8)	8 (1.3)

^1^The English items and response options have the original wording.

^2^Swedish translations in brackets.

Targeting assessments found the average total SCI score was above the scale midpoint (i.e., 16). However, scores spanned the full possible range and skewness was -0.56. Floor and ceiling effects were 9.6% and 33.5%, respectively. As shown in Table 3, considerable ceiling effects were also evident for the majority of items. Items 1–3 demonstrated a pronounced negative skew score distribution with extensive ceiling effects, i.e., indicating less sleep difficulties. Items 4–7 demonstrated a less negative skewed distribution and item 8 showed an inverted bell curve distribution with considerable floor and ceiling effects. Item level endorsement distributions across response categories are reported in Table 2.

**Table 3:** Item score distribution and inter-item correlations.

		*Score distribution, %*			*Inter-item correlations ^a^*							
Items	Mdn (q1-q3)	Ceiling	Floor	Missing	1	2	3	4	5	6	7	8
1 …how long does it take you to fall asleep?	3 (2–4)	35.8	8.0	0.3	1.00							
2 …if you then wake up during the night how long are you awake for in total?	4 (3–4)	58.4	6.3	0.2	0.51	1.00						
3 …how many nights a week do you have problems with your sleep?	4 (2–4)	52.1	12.8	0.5	0.67	0.64	1.00					
4 …how would you rate your sleep quality?	3 (2–3)	18.8	3.5	0.3	0.61	0.59	0.83	1.00				
5 …affected your mood, energy or relationships?	3 (2–3)	16.3	6.6	0.5	0.52	0.49	0.71	0.68	1.00			
6 …affected your concentration, productivity or ability to stay awake?	3 (2–3)	15.9	6.0	0.8	0.50	0.48	0.68	0.64	0.81	1.00		
7 …troubled you in general?	3 (2–3)	21.1	5.4	1.0	0.48	0.50	0.72	0.69	0.83	0.83	1.00	
8 finally… how long have you had a problem with your sleep?	3.5 (0–4)	49.4	27.8	1.3	0.57	0.60	0.83	0.77	0.65	0.63	0.70	1.00

a Polychoric correlations

The inter-item correlations should be fairly equal and at least 0.3. The average inter-item correlation was 0.65, with a range between 0.48 and 0.83. This may suggest some item redundancy (Table 3).

The eigenvalues of the empirical factors were 5.57 and 0.79 respectively. The result from the parallel analysis supported a one-factor model with corresponding 95^th^ percentile eigenvalues of 5.25 and 0.43 for the first and second factor respectively. This unidimensional model explained 72% of the total variance and the factor loadings ranged between 0.66 and 0.92. Goodness of fit statistics of the model were RMSEA = 0.022; CFI = 1.0 respectively. The internal consistency of SCI was excellent with an ordinal alpha of 0.94, which did not increase if any items were deleted. The corresponding internal consistency using traditional Cronbach’s alpha was 0.91, (Table 4).

**Table 4:** Exploratory factor analysis of the Sleep Condition Indicator (SCI).

Items	Factor loadings	Communality values (h^2^)
1 …how long does it take you to fall asleep?	0.67	0.45
2 …if you then wake up during the night how long are you awake for in total?	0.66	0.43
3 …how many nights a week do you have problems with your sleep?	0.92	0.84
4 …how would you rate your sleep quality?	0.86	0.74
5 …affected your mood, energy or relationships?	0.84	0.70
6 …affected your concentration, productivity or ability to stay awake?	0.81	0.66
7 …troubled you in general?	0.85	0.72
8 finally… how long have you had a problem with your sleep?	0.85	0.72
Eigenvalue	5.57	
Explained variance, %	69.7	
Ordinal alpha	0.94	
Cronbach’s alpha	0.91	

As hypothesized, the correlation between SCI and ISI scores was strong (r_s_ = -0.88). SCI scores also correlated strongly with PSQI scores (r_s_ = -0.85), and moderately (r_s_ = -0.50) with PSS scores. These findings support external construct validity.

**Fig 1:**
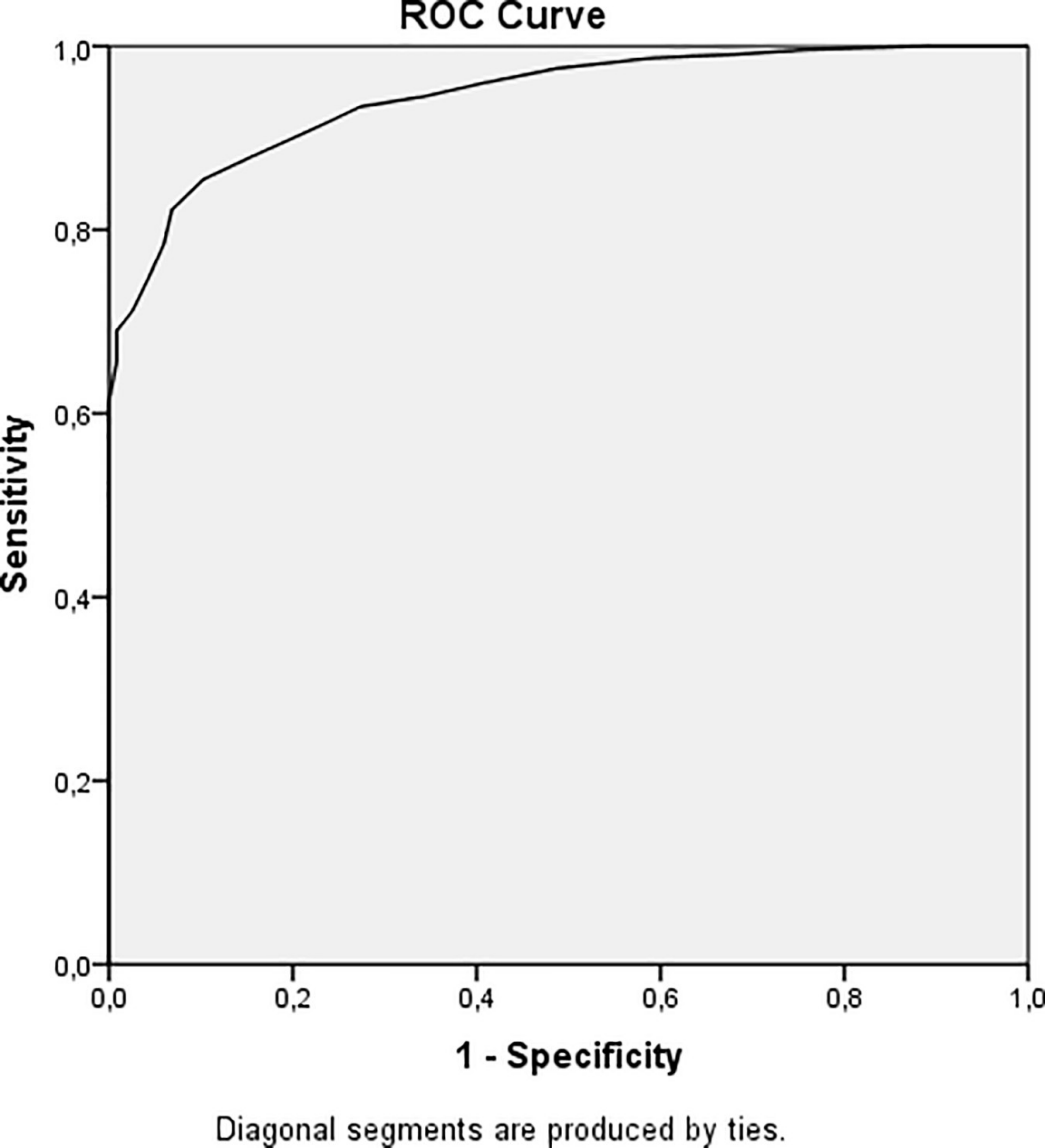
ROC curve of SCI and ISI as proxy gold standard. Area under curve = 0.94, sensitivity = 86%, specificity = 90% when J = 0.75. Scores has been reversed for the ROC-curve so that both ISI and SCI score in the same direction.

The AUROC was 0.94. A SCI score ≤16 yielded a sensitivity of 86% and a specificity of 90% (J = 0.75) (Fig 1).

## Discussion

The aim of this study was to perform a CTT based psychometric evaluation of the SCI by taking the ordinal nature of item level data into account. This is, to the best of our knowledge, the first study to address the psychometric properties of the SCI using CTT methods appropriate for ordinal level data. Our observations provide initial support for the psychometric properties of the SCI.

The SCI was considered comprehensive and easy to use according to the interview data, which also was supported by low levels of missing item responses and a high proportion of computable scale scores.

Our findings show that the SCI produces unidimensional and reliable scores in the population of this study. This is in contrast to previous studies, which have suggested multidimensionality through two-factor solutions [13, 17]. However, those studies had used a PCA and not considering the ordinal nature of data. The discrepancy could also be explained by differences between the samples or the translation. It is well known that CTT is sample dependent which implies that psychometric properties may vary between studies [34]. This illustrates the need to confirm the factor structure in further evaluations.

Targeting was generally acceptable, and similar to what was found by Wong et al. [17], who also investigated sleep in university students. However, we found relatively large ceiling effects (i.e. non-insomniacs). This is problematic from an outcome assessment point of view since the scale would be unable to detect any improvements in about one third of the sample. However, the amount of ceiling effects observed in this study is considered less concerning since the SCI primarily is a screening tool rather than an outcome assessment instrument. Furthermore, our sample did not represent individuals with sleep problems, but rather a student population in general. Considering this, the percentages of students with and without sleep problems are comparable to other studies [4, 7, 8]. There were low levels of missing item responses (0.2–1.3%), potentially indicating clarity and readability of the translated items.

CTT has been the most commonly used method for evaluating psychometric properties and relies on evidence predominantly from correlations and descriptive statistics. Strengths of the approach are familiarity, ease of adoption and use, and ability to provide tangible statistics that can be checked against existing criteria. Furthermore, problems regarding missing data and floor/ceiling effects could easily be identified by CTT [35]. In contrast to modern test theory, CTT is concerned with the instrument itself rather than the ability of the person. Therefore, CTT cannot provide assumptions about how a person may perform on a given item [36], which could be seen as a shortcoming.

### Clinical implications

Assessment of psychometric properties is mainly directed toward the conclusions that can be drawn about the attributes of people who achieve a certain score on the test[34]. Although other questionnaires for insomnia, such as the ISI, are available, the SCI adds to the current existing questionnaires by its specificity and that it is in line with the DSM-5 criteria.

Our findings provide support that the SCI is a unidimensional instrument with high reliability. It is strongly correlated with the ISI as predicted, but also with the PSQI. This adds to previous findings of other research groups and in other populations. Our CTT-based evaluation, using methods that take the ordinal nature of data into account, supports the use of SCI as a screening tool for insomnia. Since health assessment is becoming increasingly important in clinical research, trials, and practice, the instruments used for this purpose will also influence decisions about patient care and policy-making. Thus, assessment instruments need to provide us with scientifically robust results. Other recent reports on large samples suggest that the SCI is a practical tool for screening insomnia across demographic groups [6].

### Methodological considerations

Strengths of this study are the qualitative, cognitive interviews combined with the quantitative evaluation of the instrument. For the translation, a forward-backward procedure was followed, using two independent bilingual translators speaking UK English. In addition, one of the authors of the SCI was involved throughout the whole process. The analytical procedure made it possible to compare the findings with previous studies, but also to confirm the results with statistical methods more suitable for ordinal data.

The response-rate was low for the present study. While online surveys have advantages, such as the possibility to reach thousands of respondents simultaneously with low to no cost [34], they are also known to have the disadvantage of lower response rates compared to traditional mail surveys [37]. Furthermore, the primary purpose of this study was to test the psychometric properties of the SCI. As such, overall response rate is of less concern than, e.g., good coverage across possible total scores and having a sufficient absolute number of (complete) responses in relation to methods of analyses [34].

In line with our primary aim, the full 8-item version of the SCI was assessed here as part of these analyses. Previously a 7-item and 2-item version of the SCI was assessed as an outcome measure without the item, which asks about the duration of insomnia symptoms [6, 14]. This item was not expected to change over time and therefore reduced versions of the SCI were not assessed here.

Our sample consisted of university students, which implies low mean age and high educational level. In addition, the majority of the students were women. Potentially students concerned about their sleep were more inclined to participate in a survey such as this. A majority of the sample were nursing students. Due to their choice of education, these students may be more aware of and concerned about their sleep. However, there were no statistically significant differences in sleep problems between students in the different educational programs in the study.

## Conclusions

Our CTT-based psychometric testing supports the SCI as a user-friendly, unidimensional insomnia screening tool, with high internal consistency. Future research should address its psychometric properties across more diverse populations, and evaluate additional properties such as responsiveness and differential item functioning.

## Supporting information

S1 File Dataset. (SAV)
